# Association between health locus of control and perceived stress in college student during the COVID-19 outbreak: a cross-sectional study in Iran

**DOI:** 10.1186/s12888-021-03543-1

**Published:** 2021-10-26

**Authors:** Mahasty Ganjoo, Akram Farhadi, Reza Baghbani, Safieh Daneshi, Reza Nemati

**Affiliations:** 1 Department of Medical Emergencies, School of Allied Medical Sciences, Bushehr University of Medical Sciences, Bushehr, Iran; 2 Department of Health Education and Promotion, Faculty of Health, Bushehr University of Medical Sciences, Bushehr, Iran; 3 Clinical Research Development Center, Shohadaye- Khalije- Fars Hospital, Bushehr University of Medical Sciences, Bushehr, Iran

**Keywords:** Health locus of control, Perceived stress, Students, COVID-19

## Abstract

**Background:**

The COVID-19 pandemic as a global mental health crisis has affected everyone, including students. The present study aimed to determine and investigate the relationship between health locus of control and perceived stress in students of Bushehr University of Medical Sciences (southern Iran) during the outbreak of COVID-19.

**Methods:**

The present cross-sectional study examined 250 students of Bushehr University of Medical Sciences. We performed simple random sampling and utilized the demographic information form, Multidimensional Health Locus of Control scale (MHLCS) by Wallston, and Perceived Stress Scale (PSS) by Cohen to collect data. We analyzed data using the SPSS, Pearson correlation coefficient, and the hierarchical regression model with an error level of 5%.

**Results:**

The mean perceived stress was 30.74 ± 8.09, and 92.4% of the students had moderate and high stress levels. Among the components of the health locus of control, the internal health locus of control (IHLC) had the highest mean in students (27.55 ± 3.81). Furthermore, the internal health locus of control (*R* = − 0.30, *P* < 0.001) had a significant inverse relationship, with perceived stress and the chance health locus of control (CHLC) (*R* = 0.30, *P* < 0.001) had a significant direct relationship. In the final regression model, the health locus of control and all the variables predicted 22.7% of the perceived stress variation in students during the COVID-19 period.

**Conclusion:**

The results indicated that the internal health locus of control was associated with a reduction of perceived stress, and the powerful others health locus of control (PHLC) was related to its increase in students during the COVID-19 pandemic. Given the uncertain future, in the present work, universities are suggested to design web-based educational interventions alongside the curriculum to further strengthen the internal health locus of control and thus help reduce their perceived stress.

## Background

Coronavirus disease-2019 (COVID-19) is a global experience that is supreme in the history of pandemics [1, 2]. According to the WHO (World Health Organization) statistics, more than 185 million people were infected with the virus up to July 12, 2021, and more than four million died. In Iran, there are about 3 million 330 thousand sufferers and 85,397 deaths of COVID-19. Due to the high intensity of the fourth COVID-19 wave in Iran, and a very low rate of vaccination, the mortality rate is rapidly increasing [3].

Most countries have recently taken the necessary measures to change and limit citizens to control and prevent the spread of disease. These changes include the nationwide closure of universities, schools, and crowded places, the conversion of the face-to-face education system into a virtual one, and the non-accommodation of students in dormitories [4]. Due to the continuous spread of this epidemic, increasing restrictions, and social distance, people experience negative effects, including feelings of social isolation, fear, and uncertainty about the future, and mental health disorders [5]. There are many reports about the negative effects of this pandemic on the mental health of various social groups [6]. Even though young people have had less physical vulnerability and mortality in facing this virus [7], they are the most vulnerable people in terms of psychological damages caused by this pandemic [8–10]. Higher psychological vulnerability in students is due to serious changes in student life, lower interactions with groups of friends and peers, challenges of e-learning, concerns about the quality of education, concerns regarding the future of work and education, and challenges with family due to long time presence at home [10–12]. Among these negative consequences in students’ mental health is an increase in their perceived stress, which is somehow related to an individual’s mental judgment of life events [12, 13].

According to Lazarus and Folkman (1984) (p. 19), “psychological stress is a particular relationship between the person and the environment that is appraised by the person as taxing or exceeding his or her resources and endangering his or her well-being” [13]. Perceived stress is strongly associated with anxiety [14], and depressive symptoms [15]. Despite the complexity of the relationship between perceived stress and mental health, their direct cause-and-effect relationship is not clear yet [15]. There are reports about the prevalence of high perceived stress in students in the United States (71%) [9], Turkey (52%) [16], France (61.6%), Saudi Arabia (35%) [17], and China (41.1%) [18] during COVID-19. Also in a study in Bangladesh evaluating the psychosocial impact of COVID-19 on students, Hossain et al. (2021) found that the coronavirus pandemic had caused a number of psychological problems, including stress and anxiety, in students [19]. On the other hand, Aslan et al. (2020) in their study aimed at examining perceived stress among students in Turkey during the COVID-19 pandemic, found a 71% prevalence of high levels of perceived stress, and clinical signs of anxiety were reported in more than half of the students (52%) [16].

Considering the potential and serious risks of the COVID-19 pandemic for various aspects of students’ mental health, including their perceived stress, and the continuation of the current online education process, it is necessary to examine factors associated with this perceived stress [12, 20]. According to studies, a high percentage of individuals’ health in the COVID-19 pandemic crisis is affected by their behavior and beliefs [21]. Among the different behaviors and dimensions of health, the health control center is an important variable in the individuals’ health behaviors [22].

The theory of locus of control was introduced in 1966 by Rotter, which was derived from Bandura’s social learning theory. The locus of control expresses the individuals’ perception of their abilities to exercise control over the environment, and it introduces two types of internal and external control axes [23]. Wallston et al. (1978) successfully introduced and implemented the original idea of Rotter in the field of health [22].

Health locus of control refers to the belief that individuals’ health relies on the control of internal factors (including the individual’s behaviors and actions) or other factors (chance and health-care staff, such as doctors and nurses). Therefore, people believe that they themselves, other powerful people, and chance can affect their health condition [22]. People with higher IHLC have more positive health habits and motivation to stay healthy [24]. Some studies during the COVID-19 pandemic indicate that higher levels of IHLC are associated with better mental health [20], reduced risk of suicide [25], and improved well-being [24].

Also, Khumalo et al. (2019) examined the relationship between the source of control and depression among undergraduate students in Botswana. The findings supported that students who believed in having control over their life events were less likely to experience depressive symptoms, while students who believed their lives were controlled by luck or others experienced higher depressive symptoms [26]. Millman et al. (2017) reported that people who were at high risk for psychosis experienced more social stress and also considered sources of external health control, such as external factors and luck, effective in their health. Other results of their study indicate a strong relationship between external health control sources and social stress [27].

Therefore, due to the increasing prevalence of psychological disorders during the coronavirus pandemic [5, 18], and the limitations of studies, it is important to assess the individuals’ tendencies to each component of the health locus of control. During the present study in Iran, the teachings were online at all universities, except for a limited number of clinical students in the fields of medical sciences (internships). Moreover, the professors and staff of the universities worked through teleworking.

### Aim of the study

The aim of this study was to determine the relationship between health locus of control and perceived stress in students of Bushehr University of Medical Sciences (southern Iran) during the COVID-19 outbreak.

## Methods

### Design, settings, and participants

The present cross-sectional study contained the statistical population consisting of all students of Bushehr University of Medical Sciences (southern Iran). The study was conducted during the coronavirus outbreak from 20 January 2021 to 22 February 2021. We performed simple random sampling among students based on the student registration system. Inclusion criteria of the study were as follows: being a student at any grade of education at Bushehr University of Medical Sciences (southern Iran); lack of COVID-19 during the study, and having informed consent to enter the study. Exclusion criteria: a history of mental disorder under treatment in self-reports, and incomplete questionnaires. After a random selection of the students’ names, the liaison education expert received an electronic link for completing the questionnaires in each faculty. The link would be sent to the students via WhatsApp after obtaining their informed consent. The sample size was obtained as 221 considering an effect size of Cohen’s *ρ* = 0.2, Alpha error of 0.05, and test power of 85% in a two-way Pearson correlation test using PASS 15. Considering the 15% probability of loss, the sample size was considered as 255.

### Data quality control

The data of the completed questionnaires were placed on the server of the website for the online questionnaire, for which the researcher had access. Therefore, there was no need for students to email the questionnaire information. After completing and registering the questionnaire, the website prevented the completion of a questionnaire more than once according to its capabilities and detection of the same IPs.

### Data collection procedure and instruments

We used the demographic and clinical data collection form, the Multidimensional Health Locus of Control scale (MHLCS) by Wallston, and the Perceived Stress Scale (PSS) by Cohen to collect data.

Demographic and clinical information form included age, gender, marital status, academic year, current residence, family income, employment at the hospital, following coronavirus news, up-to-date information about coronavirus, death of relatives and friends due to coronavirus, feelings when quarantined at home, infection with coronavirus, history of psychiatric illness, concern about coronavirus infection, family employment at the hospital, history of coronavirus infection in family, relatives, and friends, living in high-risk areas in terms of the spread of coronavirus, and taking adequate measures against the coronavirus. All the information was collected through self-report.

#### Multidimensional health locus of control scale (MHLCS)

The Multidimensional Health Locus of Control scale (MHLCS) was designed by Wallston et al. [22] in 1978 to determine the individuals’ health locus of control. The questionnaire evaluates three domains, namely powerful others health locus of control (PHLC), internal health locus of control (IHLC), and Chance health locus of control (CHLC). The powerful others health locus of control (PHLC) believes that the health of a person is affected by other people. The internal health locus of control (IHLC) includes individuals’ degrees of belief where the internal factors and behaviors are responsible for their illness and health. The Chance health locus of control (CHLC) includes individuals’ degrees of believing that their health depends on chance, fortune, and destiny. This questionnaire consists of 18 items on a six-point Likert scale from strongly disagree [1] to strongly agree [6]. The options of strongly disagree and strongly agree are scored 1 to 6. Each subscale receives a score of 6 to 36. Three subscales are not added and estimated independently [22]. The validity and reliability of this questionnaire were investigated by Moshki et al. (2007) in Iran. The Cronbach’s alpha coefficients were 0.68, 0.66, and 0.72 for the internal health locus of control, Chance health locus of control, and powerful others health locus of control, respectively [28].

#### Perceived stress scale (PSS)

The Perceived Stress Scale (PSS) was designed by Cohen et al. [29] in 1983. It consists of 14 questions on a five-point Likert scale to assess the general perceived stress in the past month as well as thoughts and feelings about stressors, controlling, overcoming, or coping with the experiencing stress. The scores are inversely calculated in positive questions [4–7, 9, 10 and 13]. On this scale, the minimum perceived stress pressure score is 0 and the maximum is 56. A higher score means higher perceived stress. If the average score is from 0 to 14, the level of stress is low, 15 to 28 refer to moderate stress, and 29 to 56 represent the high perceived stress [29, 30]. The Cronbach’s alpha coefficient was used to calculate the reliability of the questionnaire in the study of Mohammadi et al. (2019), and it was obtained as 0.83, indicating the high reliability of the questionnaire [31].

### Ethical consideration

The present study was reviewed and approved by the ethics committee of Bushehr University of Medical Sciences with an ethics code (IR.BPUMS.REC.1399.141). The participants were ensured that their participation in the study was voluntary and their names would not be included in the questionnaire for the confidentiality of the individuals’ information. Informed consent was also obtained from the participants under the Helsinki Accords.

### Data analysis

We analyzed data using the Statistical Package for the Social Sciences (SPSS 22) (SPSS Inc., Chicago, IL, USA). For descriptive analysis of data, we used descriptive statistics such as mean, and standard deviation for quantitative variables, and frequency and percentage for qualitative variables. The normality of data distribution was confirmed using Kolmogorov-Smirnov statistical test. T-test and ANOVA test were used to compare the mean of stress, and Pearson correlation coefficient was used and to examine the relationship between health locus of control and perceived stress. Using a hierarchical regression model and including all the demographic and clinical variables in the study, we examined the role of health locus of control on predicting perceived stress in students. The significance levels of all analyses were considered as 0.05.

## Results

### Socio-demographic characteristics

In the present study, 23 out of 273 questionnaires were excluded owing to non-completion, and finally, questionnaires of 250 students were included in the study (response rate = 91.5%). The mean age of the students participating in the study was 22.23 ± 3.60 in the age range of 18 to 45 years. The majority of the participants were women (58.4%), single (90.8%), living in a father’s house with their family (88%), with a feeling of bored due to being quarantined at home (41.2%), following coronavirus news most often (40.4%), no personal history of coronavirus (83.2%), having a history of coronavirus in friends and relatives (86%), no history of the death of relatives and friends due to coronavirus (61.6%), and being concerned about coronavirus infection (62.8%). Among the participating students, 90.8% reported no history of psychological problems under treatment, and 87.2% believed that their measures taken to prevent the coronavirus, were sufficient and appropriate (Table 1).

**Table 1:** Demographic characteristics and comparison of Perceived Stress score among characters in each group (*n* = 250)

Variables	Number (%)	Mean (SD)	***P*** value	Variables	Number (%)	Mean (SD)	***P*** value
Gender				Employment at the hospital			
Male	104 (41.6)	24.97 (8.61)	0.203	Yes	16 (6.4)	23.75 (8.08)	0.310
Female	146 (58.4)	26.29 (7.69)		No	234 (93.6)	25.88 (8.09)	
Marital Status				**Following coronavirus news**			
Single	227 (90.8)	26.03 (8.17)	0.114	Sometimes	99 (39.6)	26.48 (7.27)	0.135
Married	23 (9.2)	23.22 (7.06)		Mostly	101 (40.4)	24.50 (8.02)	
Academic year				Always	50 (20.0)	26.78 (9.53)	
First year	43 (17.2)	22.93 (7.94)	0.013^*^	**up-to-date information about coronavirus**			
Second year	75 (30.0)	25.07 (8.93)		Yes	216 (86.4)	25.20 (8.12)	0.007^*^
Third year	57 (22.8)	27.28 (7.95)		No	34 (13.6)	29.21 (7.13)	
Fourth year	45 (18.0)	25.93 (6.92)		**Death of relatives and friends due to coronavirus**			
Fifth year	13 (5.2)	28.54 (5.06)		Yes	96 (38.4)	26.52 (8.69)	0.232
Sixth year	14 (5.6)	26.00 (7.18)		No	154 (61.6)	25.26 (7.69)	
Seventh year	3 (1.2)	37.67 (7.57)		**History of psychiatric illness**			
Current residence				Yes	23 (9.2)	29.52 (6.93)	0.019^*^
Personal home	220 (88.0)	25.52 (7.94)	0.243	No	227 (90.8)	25.36 (8.12)	
Dormitory	30 (12.0)	27.37 (9.15)		**Concern about coronavirus infection**			
Family income				Yes	157 (62.8)	25.80 (8.25)	0.75
Less than $50	32 (12.8)	26.28 (7.73)	0.042^*^	No	21 (8.4)	26.81 (8.36)	
50 to $100	45 (18.0)	27.11 (9.22)		I’m somewhat worried	72 (28.8)	25.32 (7.76)	
$ 100 to $200	82 (32.8)	26.90 (7.90)		**Family employment at the hospital**			
Over $ 200	91 (36.4)	23.84 (7.56)		Yes	93 (37.2)	25.30 (8.73)	0.507
Feelings when quarantined at home				No	157 (62.8)	26.01 (7.72)	
Impatient	103 (41.2)	25.05 (7.67)	< 0.001^*^	**History of coronavirus infection in family, relatives, and friends**			
Worried	25 (10.0)	28.80 (7.45)		Yes	215 (86.0)	25.41 (7.85)	0.110
Angry	27 (10.8)	30.48 (8.92)		No	35 (14.0)	27.77 (9.37)	
Sense of security	55 (22.0)	25.64 (8.77)		**Infection with coronavirus**			
Other	40 (16.0)	22.58 (6.16)		Yes	42 (16.8)	25.36 (9.42)	0.766
Taking adequate measures against the coronavirus				No	208 (83.2)	25.82 (7.83)	
Yes	218 (87.2)	25.38 (8.14)	0.061	**Living in high-risk areas of coronavirus**			
No	32 (12.8)	28.25 (7.45)		Yes	104 (41.6)	27.03 (8.77)	0.034*
				No	146 (58.4)	24.83 (7.48)	

*. ***p*-value < 0.05**

### Comparison of perceived stress score between demographic variables

As you can see in Table 1, academic year, income level, feeling quarantined at home, up-to-date information about coronavirus, a history of mental illness, and living in high-risk environments have a significant relationship with perceived stress levels.

The results of a one-way analysis of variance showed that there was a significant difference between the perceived stress scores between the academic years (*P* = 0.013). Scheffe post hoc test showed that the perceived stress score in seventh-year students was significantly higher than first-year students (*P* = 0.034).

The results of a one-way analysis of variance showed that there was a significant difference between the perceived stress between the 4 groups of families’ incomes (*P* = 0.042). In the LSD post hoc test, the average stress score among families earning between $50 and $100 (27.11 ± 9.22) and families earning more than $200 per month (23.84 ± 7.56) was significant (*P* = 0.026). Also, there was a significant difference (*P* = 0.013), between families whose income was between $100 and $200 (26.90 ± 7.90) and families whose income was more than $ 200 per month.

The results of a one-way analysis of variance showed that there was a significant relationship between perceived stress and the feeling of quarantined (*P* < 0.001). Scheffe post hoc test showed that there was a significant relationship between the perceived stress score of people who experienced “other emotions” with the experience of feeling anxious (*P* = 0.048) and feeling angry (*P* = 0.003). The findings also showed that people who felt “angry” and “worried” at the time of quarantine had more perceived stress.

People who kept their coronavirus information up to date (25.20 ± 8.12) had significantly less perceived stress than those who did not keep their information up to date (29.21 ± 7.13) (*P* = 0.007).

People with a history of psychological disorders had the perceived stress (29.52 ± 6.93) significantly higher than those who did not have a history of psychological disorders (25.36 ± 8.12) (*P* = 0.019).

People who lived in high-risk areas of the coronavirus (27.03 ± 8.77) perceived stress as significantly higher than those who did not live-in high-risk areas of the coronavirus (83.83 ± 7.48) (*P* = 0.034).

### Health locus of control & perceived stress level

Among the components of the health locus of control, the internal health locus of control (27.55 ± 3.81), the powerful others health locus of control (21.27 ± 5.21), and the internal health locus of control (16.78 ± 5.17) had the highest mean respectively. The mean perceived stress was 25.74 ± 8.09. According to the results of the present study, 7.6% showed low stress, 58.4% had moderate stress, and 34% had high stress. It was indicated that 92.4% of the students had moderate and high stress levels (Table 2).

**Table 2:** Mean and standard deviation of health locus of control and perceived stress (*n* = 250)

Variable	Mean	Standard deviation
**Internal Health Locus of Control**	27.55	3.81
**Powerful Other Locus of Control**	21.27	5.21
**Chance Locus of Control**	16.78	5.17
**Perceived Stress**	25.74	8.09

### Correlation of health locus of control and perceived stress

Table 3 presents the correlation between the health locus of control and perceived stress. According to the findings, the internal health locus of control (*R* = − 0.30, *P* < 0.001), and the chance health locus of control (*R* = 0.30, *P* < 0.001) had a significant correlation with the perceived stress. The correlation between the powerful others’ health locus of control and perceived stress was not significant (Table 3).

**Table 3:** Correlation matrix of health locus of control and perceived stress

**Variable**	**1**	**2**	**3**	**4**
**1- Internal Health Locus of Control**	1	–	–	–
**2- Powerful Other Locus of Control**	0.10	1	–	–
**3- Chance Locus of Control**	−0.12	0.33^a^	1	–
**4- Perceived Stress**	−0.30^a^	0.09	0.30^a^	1

^a^Correlation is significant at the 0.01 level

### The predictive role of the health locus of control and demographic and clinical variables on perceived stress

Table 4 presents the correlation between the health locus of control and perceived stress in students based on the hierarchical regression model. In the first model, the two dimensions of internal health control and chance-related control focus predict 16.6% of perceived stress in students. After entering the demographic variables (family’s income) in model 2, the power of predicting perceived stress increased by 2.9%. In the final model, by adding the variables relating to clinical status and coronavirus-related conditions, 3.3% is added to the prediction power. In the final model, all the variables predict 22.7% of the perceived stress changes in students during the coronavirus outbreak. Among the demographic and clinical variables, included in the model as confounders, only the amount of family’s income (B = − 0.137, *P* = 0.019) showed a significant correlation (Table 4).

**Table 4:** The Predictive Role of the Health Locus of Control and Demographic and Clinical Variables on Perceived Stress^d^

Model	R	R Square	Adjusted R Square	Std. Error of the Estimate	Change Statistics					***P*** value
					R Square Change	F Change	df1	df2	Sig. F Change	
**1**	0.407^a^	0.166	0.159	7.426	0.166	24.532	2	247	< 0.001	< 0.001
**2**	0.441^b^	0.194	0.181	7.328	0.029	4.344	2	245	0.014	< 0.001
**3**	0.477^c^	0.227	0.201	7.236	0.033	2.562	4	241	0.039	< 0.001

^a^Predictors: (Constant), IHLC, CHLC

^b^Predictors: (Constant), IHLC, CHLC, Academic year, Family income

^c^Predictors: (Constant), IHLC, CHLC, Academic year, Family income, Feelings when quarantined at home, up-to-date information about coronavirus, History of psychiatric illness, Living in high-risk areas of coronavirus

^d^Dependent Variable: Perceived Stress

## Discussion

The COVID-19 pandemic indicated the need for timely diagnosis and treatment of psychological aspects of epidemics as phenomena with psychological effects. The aim of this study was to determine the relationship between health locus of control and perceived stress in students of Bushehr University of Medical Sciences (southern Iran) during the COVID-19 outbreak.

### Prevalence of perceived stress in students

The results of the present study indicated that a high percentage of students (92.4%) had moderate and high stress levels. Considering the participants’ average age and since most students were in their youth, they sought to explore identity, strived to achieve independence, and had different roles to play [32]. Moreover, given other stresses, high levels of perceived stress might be caused by holding online exams and relevant strictures, extensive and online curricula, constantly changing programs for internships in clinical courses, designating the only teaching hospital affiliated with the university as a hospital dedicated to the treatment of patients with COVID-19, and concerns about coronavirus infection according to demographic data. Most of the participants in the present study were female. According to similar reports and studies, high levels of stress in women were attributed to various factors, such as hormonal changes and the expression of feelings and thoughts about their social status that could be other causes of higher perceived stress in the participants [33, 34].

According to the results, increasing the education years enhanced the students’ perceived stress. In other words, increasing the education years enhanced the students’ mental concerns regarding the uncertainty of their academic and professional future owing to the coronavirus pandemic [16].

Recent studies also confirmed the results of the present study. Wang et al. (2021) and Moghanibashi-Mansourieh et al. (2020) found that there was a direct and significant correlation between education level and stress and anxiety level during the COVID-19 pandemic [35, 36]. According to a study in China, the higher prevalence of mental symptoms was reported among individuals with higher education, which was probably due to their high self-awareness about their health [37].

Furthermore, all theoretical and practical courses for freshmen were online and they were not present in high-risk and clinical places. However, since the only teaching hospital under the supervision of Bushehr University of Medical Sciences was allocated as a reference hospital for the hospitalization and treatment of such patients during the coronavirus epidemic, clinical students had to attend the hospital to pass their clinical courses, which could be effective in increasing their stress.

Another result of the study indicated the inverse and negative correlation of family income and perceived stress. As the family income decreased, perceived stress levels increased. Also, the present study showed that after entering the demographic and clinical variables, 6.1% was added to the predictive power of perceived stress, which among the demographic variables, only the amount of family’s income had the power to predict the perceived stress. Due to the closure of some self-employed businesses, and the lack of formal government support for informal jobs to compensate for coronavirus during the pandemic, students with guardians working in such jobs with no sufficient financial resources, were in dire financial straits. Hence, they had serious difficulty even in the provision of virtual education tools and facilities such as smartphones, tablets, or the cost of the Internet. They also did not officially receive support from the university, thereby increasing their stress.

Reports indicate that families with children who were studying (at school or university) were experiencing a financial crisis in the COVID epidemic. Aslan et al. (2020) also found that declining income leads to increased perceived stress levels [16], while Chen et al. (2021) reported that high-income families with school children experienced more stress than low-income families with school children [38]. One of the reasons for the discrepancy between the results of their study and the present study is the society under study. In their study, Chen et al. surveyed families with children from preschool to high school. On the other hand, high-income families can be stressed for their children due to sudden exposure to educational problems as well as feeling additional responsibilities of being involved in their children’s education. But low-income families are more concerned about their family’s basic needs, such as food, clothing, and shelter, than arranging their children’s education and planning home-based education programs for their children. In the present study, the university student population has been investigated. The difference is that university students are less dependent on family planning programs than their school-age children.

The results showed that people who kept their scientific knowledge about coronavirus up to date experienced lower perceived stress levels. The World Health Organization (WHO) also states that daily follow-up of bad news about coronavirus is one of the factors that increase the level of stress and anxiety [39]. The statement also emphasizes that information about this disease should be available to everyone through reliable sources. In the present study, however, following the COVID-19 news had no effect on students’ stress levels. Explaining this finding, it can be said that due to the fact that the study population of the present study is students of the University of Medical Sciences, students have more access to credible references to provide news and information about the coronavirus through professors and course resources than the general public. The results of a study by Teshome et al. (2021) also showed that people who do not keep their information about coronavirus up to date experience twice as much stress levels as people who keep their information up to date [40]. These results confirm the results of the present study.

The results of the present study also showed that people with a history of psychological disorders experience more perceived stress. Explaining these results, it can be said that people who have previous psychological problems are usually less able to cope with stressful situations [41]. Similarly, Oppenauer et al. (2021) reported in their study that stress, anxiety, and depressive symptoms associated with coronavirus were four times more common in patients with psychological disorders [42].

Another factor in the prevalence of perceived stress is living in high-risk areas due to the prevalence of COVID-19, where living in these areas increases the level of perceived stress. The high prevalence of coronavirus throughout the country, including Bushehr province, at the time of sampling, leading to cases of hospitalization and mortality of friends, more restrictions on quarantine regulations and traffic bans, as well as public recreational places being closed, all can justify some of the meaningful increase of perceived stress.

### The correlation between health locus of control and perceived stress

The present results indicated that the health locus of control (internal health locus of control and chance health locus of control) had a significant correlation with perceived stress in students. Hence, the perceived stress showed a significant inverse correlation with internal health locus of control and a significant direct correlation with chance health locus of control. Karkoulian et al. (2016) studied gender perspective on work-life balance, perceived stress, and locus of control. They found that perceived stress had an inverse and significant correlation with the internal health locus of control [43]. The results were consistent with the results of the present study. Other results of the study by Karkoulian et al. indicated the inverse and significant correlation of perceived stress and external health locus of control, such as powerful others and chance health locus of control [43]. The results were inconsistent with the results of the present study because the study by Karkoulian et al. examined bank employees before the coronavirus pandemic, however, the present study investigated medical students in the coronavirus pandemic, which could affect the results. The importance of self-care and observing the health protocols by individuals was constantly emphasized by the mass media during the coronavirus pandemic. Moreover, the participants mostly followed the health protocols to prevent coronavirus. Owing to their field of study and knowledge about health aspects, they always emphasized their internal health locus of control and placed less emphasis on locus of control by others and chance. Hence, they were different from other groups.

In the present study, the internal health locus of control, among the three components of the health locus of control, obtained the highest mean in the participants. The prevalence of psychological disorders during the coronavirus outbreak was considered in students [18] along with their tendency with psychological disorders to powerful others health locus of control [25]. Moreover, the source of internal health locus of control was associated with a better performance in health instructions [44]. Thus, the higher mean of internal health locus of control in students probably indicated the appropriate activity of various media in informing about the announcement of coronavirus health protocols and their strong emphasis on the impact of self-care on the prevention of the virus. Furthermore, their education in medical science was emphasized along with higher knowledge about health care, as well as an emphasis on adhering to individual protocols.

### Limitations and future research directions

Like most research studies, the present study had also limitations. Since the study was cross-sectional, it is not possible to conclude a cause-and-effect relationship between the variables. Therefore, we recommend conducting longitudinal studies to eliminate this limitation. The data of the present were collected by a self-report. Due to the special circumstances during the COVID-19 pandemic, the information could be affected by a self-reporting bias. Further qualitative studies can be helpful to achieve more complete information. The existence of a dependent variable was another limitation of the study. We suggest examining other psychological aspects in future studies. Since the study investigated the students at only a university of medical sciences, there was a limitation in the generalizability of the results.

## Conclusion

The research results indicated that the health locus of control was considered as an important factor in students’ mental health such as perceived stress during the COVID-19 pandemic. Hence, the internal health locus of control was associated with decreased perceived stress and the powerful others health locus of control. In such a circumstance, where many students have moderate and high levels of stress and the status of the pandemic and student education conditions at universities in the future are unknown and uncertain, strengthening the internal health locus of control can have a protective effect against the reduction of adverse consequences such as perceived stress. To reduce the perceived stress in students, we can use counseling and mental health centers of universities, mental health peer-assistance groups among students, and educational groups in colleges. We can also design web-based educational interventions based on existing cultural, values, and religious beliefs in the Iranian society and further strengthen their health locus of control.

## Data Availability

The datasets used and/or analyzed during the current study are available from the corresponding author on reasonable request.

## Abbreviations

MHLC: Multidimensional Health Locus of Control
PHLC: Powerful others Locus of Control
IHLC: Internal Health Locus of Control
CHLC: Chance Locus of Control
PSS: Perceived Stress Scale
IRB: Institutional Review Board
